# Domestic use of pesticides during early periods of development and risk of testicular germ cell tumors in adulthood: a French nationwide case-control study

**DOI:** 10.1186/s12940-021-00795-y

**Published:** 2021-10-28

**Authors:** Aurélie M. N. Danjou, Olivia Pérol, Astrid Coste, Elodie Faure, Rémi Béranger, Helen Boyle, Elodie Belladame, Lény Grassot, Matthieu Dubuis, Johan Spinosi, Liacine Bouaoun, Aude Fléchon, Louis Bujan, Véronique Drouineaud, Florence Eustache, Isabelle Berthaut, Jeanne Perrin, Florence Brugnon, Barbara Charbotel, Joachim Schüz, Béatrice Fervers, For the TESTIS study group

**Affiliations:** 1 Environment and Lifestyle Epidemiology Branch, International Agency for Research on Cancer/World Health Organization, 150 cours Albert Thomas, 69372 Lyon, Cedex 08 France; 2 Département Prévention, Cancer et Environnement, Centre Léon Bérard, Lyon, France; 3 INSERM UMR1296 Radiation: Defense, Health, Environment, Lyon, France; 4 Université Paris-Saclay, UVSQ, Univ. Paris-Sud, INSERM, Gustave Roussy, Équipe “Exposome et Hérédité”, CESP, 94805 Villejuif, France; 5 IRSET (Institut de Recherche en Santé, Environnement et Travail), UMR S 1085, INSERM, EHESP, CHU Rennes, Rennes University, Rennes, France; 6 Department of Medical Oncology, Centre Léon Bérard, Lyon, France; 7 Direction Santé Travail, équipe associée à L’UMRESTTE (UMR T 9405 Université Lyon 1, IFSTTAR), Santé publique France, Lyon, France; 8 DEFE (Développement Embryonnaire, Fertilité, Environnement) INSERM 1202 Universités Montpellier et Toulouse 3, CECOS Hôpital Paule de Viguier, CHU de Toulouse, Toulouse, France; 9 Fédération Française des CECOS, Paris, France; 10 CECOS Hôpital Cochin, Paris, France; 11 Laboratoire d’Histologie, Biologie de la Reproduction, CECOS Hôpital Tenon, Paris, France; 12 APHP Sorbonne University, Paris, France; 13 CNRS, IRD, IMBE, Avignon University, Aix Marseille University, Marseille, France; 14 Centre Clinico-Biologique d’AMP-CECOS, AP-HM La Conception University Hospital, Marseille, France; 15 CHU Clermont-Ferrand, CHU Estaing, AMP, CECOS, Clermont-Ferrand, France; 16 IMOST, INSERM U1240, Faculté Médecine Clermont-Ferrand, Clermont-Ferrand, France; 17 UMRESTTE, UMR T 9405, IFSTTAR, Lyon 1 University, Lyon University, Eiffel University, Lyon, France

**Keywords:** Case-control study, Domestic use, Epidemiology, Environment, Pesticides, Testicular germ cell tumor

## Abstract

**Background:**

Testicular germ cell tumours (TGCT) are the most frequent cancers in young men in developed countries and their incidence rate has doubled worldwide over the past 40 years. Early life exposures to pesticides are suspected to increase TGCT risk. Our research aimed at estimating adult TGCT risk associated with parental domestic use of pesticides during early periods of child development.

**Methods:**

We conducted a case-control study of 304 TGCT cases, aged 18–45 years old, recruited in 20 French university hospitals, and 274 controls frequency-matched on hospital and birth year. Participants’ mothers provided information on their domestic use of pesticides from 1 year before start of pregnancy to 1 year after their son’s birth, for gardening activities, treatment of indoor plants, pets, wood and mold, and pest control. Odds ratios (OR) for TGCT (overall and by histological subtype) and 95% confidence intervals (CI) were estimated using conditional logistic regression.

**Results:**

Prevalence of reported domestic use of pesticides was 77.3% for insecticides, 15.9% for fungicides and 12.1% for herbicides. While no association was found for any use of insecticides (OR = 1.27, CI = 0.80–2.01) or herbicides (OR = 1.15, CI = 0.67–2.00), elevated risks of TGCT overall (OR = 1.73, CI = 1.04–2.87) and non-seminoma subtype (OR = 2.44, CI = 1.26–4.74) were observed for any use of fungicides. When specific purposes were examined, using fungicides and/or insecticides for woodwork (OR = 2.35, CI = 1.06–5.20) and using insecticides on cats and dogs (OR = 1.95, CI = 1.12–3.40) were associated with increased risk of non-seminoma subtype. We found no association for seminoma subtype.

**Conclusions:**

Although recall bias may partially explain the elevated ORs, our study provides some evidence of a positive association between domestic use of pesticides during early periods of development, particularly fungicides and risk of adult TGCT and non-seminoma. Given the common domestic use of pesticides in France, further research on TGCT risk is warranted.

**Supplementary Information:**

The online version contains supplementary material available at 10.1186/s12940-021-00795-y.

## Background

While overall testicular germ-cell tumours (TGCT) are a rare cancer, accounting for 1% of all neoplasms, these are the most common cancer occurring in young men aged 15 to 45 years in developed countries, in particular among Caucasian populations. Their incidence rate has importantly increased over the past 40 years [1–3]. TGCT in young men represent 94–98% of testicular cancers [1, 4]. These are preceded by germ cell neoplasia in situ (GCNIS), and comprise two main histological subtypes, seminomas (SE) and non-seminomas (NS), which also include mixed tumours.

Only few risk factors for TGCT have been established to date – i.e. genetic predisposition and previous history of testicular cancer – and alone they do not explain the increase in TGCT incidence rates over time, nor the differences observed between population groups and geographic regions [5, 6]. Pre- and perinatal factors are putative risk factors because GCNIS-related TGCT occur relatively early in life and have been found associated with other male reproductive disorders starting during foetal life, including congenital malformations (cryptorchidism and hypospadias), which could be signs of a common testicular dysgenesis syndrome (TDS) [7]. Environmental exposures to pesticides with endocrine disrupting properties have been suggested to be determinants of TGCT [8, 9].

Pesticides are defined as “any substance or mixture of substances of chemicals or biological ingredients intended for repelling, destroying or controlling any pest, or regulating plant growth” [10]. Multiple sources contribute to the overall pesticide exposure, including occupation, residence in the vicinity of agricultural areas, contaminated food intake and drinking water, and domestic use (indoor, in the garden or on pets). Domestic use of pesticides appears common and a non-negligible source of exposure in the general population. In 2014, 75% of the French households interviewed reported having used pesticide products at home at least once during the preceding year [11]. In a study on pesticide hair contamination in pregnant women, approximately one third of the most concentrated pesticides detected were pesticides with domestic usage [12]. So far, very few studies have examined the association between domestic use of pesticides and risk of TGCT. The use of pesticides during gardening activities and household use of insecticides have been found associated with increased risks of TGCT and NS in studies investigating exposures during adulthood [13–15]. To our knowledge, no study has focused on early periods of development, namely preconception, pregnancy and early infancy, whereas the first trimester of pregnancy has been identified as the masculinization programming window [16–19]. Domestic use of pesticides during pregnancy or around birth has been found to be associated with other cancers in the offspring, especially childhood leukemia and brain tumors [20, 21].

Here we report results from a French nationwide case-control study designed to investigate whether early life exposures to environmental risk factors and in particular pesticide use may increase the risk of TGCT in young men. This research aimed at estimating the risk of TGCT in adulthood associated with domestic use of pesticides during early periods of development, covering the period ranging from 1 year before start of pregnancy to 1 year after birth. The research further investigated the association according to TGCT histological subtypes.

## Methods

### Study design

A multicenter prospective case-control study was conducted between January 2015 and April 2018 in 20 out of the 23 university hospital centers in Metropolitan France. The study protocol has been described previously [22]. Briefly, the study included patients diagnosed with primary GCNIS-related TGCT, aged 18 to 45 years, and referred for semen preservation prior to TGCT treatment to the regional sperm banks located in the university hospitals affiliated with the French national network of study and preservation centers for eggs and semen (*Fédération Française des Centres d’étude et de conservation des oeufs et du sperme*, CECOS). Recruitment of TGCT cases had to have been performed within 12 months of diagnosis. Two groups of controls, with no personal history of testicular cancer or cryptorchidism as it may be associated with higher risk of TGCT occurrence, were recruited and frequency-matched to cases on year of birth (+/− 3 years) and hospital center. Group A controls were sperm donors and partners of women consulting for fertility disorders, with normal sperm production (total count ≥39 M sperm cells per ejaculate) and recruited in CECOS and in assisted reproduction treatment (ART) centers respectively. Group B controls were partners of women treated for a pathological pregnancy in specialized maternity clinics equipped with maternal and neonatal intensive care units, adjacent to hospital centers. Referral and recruitment of cases and both groups of controls were regional. Participants born in Metropolitan France were eligible.

After written consent, participants received a handout to prepare for the interview. In addition, they were asked to provide written permission to contact their biological mother or the closest relative in case the mother was not available. Trained investigators (IPSOS Company) conducted a 90-min telephone interview with the participants, blinded to the case-control status, and using a structured, pretested and computer assisted questionnaire [23]. Upon agreement of the participant, the mother/relative was invited to participate in the study by telephone interview, using the same procedures as for the participants.

Data collected from participants included residential history from birth onwards, lifetime occupational history, including workplace addresses and information on specific exposures for each job (pesticides, solvents, metals and welding fumes, and plastic), socio-economic status, birth characteristics, medical history and lifestyle factors (smoking status and drug use). Participants’ mothers/relatives who consented to participate provided similar information as well as additional data covering pregnancy and postnatal periods (treatments, age, morphology, breastfeeding), and reported the occupational history of the father from 1 year before conception to when the son turned 17 years old. Moreover, the questionnaire comprised items related to domestic use of pesticides, by participants at puberty and young adulthood, and by mothers from 1 year before start of pregnancy to 1 year after the son’s birth and at puberty. Sixty percent (*N* = 698) of the participants provided two blood specimens at inclusion that have been frozen and stored for latter analysis.

Both participants and mothers/relatives provided written informed consent prior to entry in the study. Participants were compensated for answering the questionnaire (20€ in gift voucher) and providing blood samples (additional 20€). The study received ethical approval from the French Ethics Committee (ref. no. A14–94), the French national agency for medicines and health products safety (ref. no. 140184B-12) and the IARC Ethics Committee (ref. no. 14–26), and was declared to the *Commission nationale Informatique et Libertés* (MR-001, ref. no. 2016–177).

### Ascertainment of GCNIS-related TGCT cases

TGCT cases were histologically confirmed by review of pathology reports and serum tumor markers (84%) by a TGCT expert (HB), and classified as seminomas or non–seminomas (embryonal carcinoma, choriocarcinoma, yolk sac tumor, teratoma and mixed germ cell tumor) according to the International Classification of Disease for Oncology (ICD-O) and the WHO classification of tumors of the urinary system and male genital organs [4, 24]. Patients with testicular cancer not originating from GCNIS were excluded (spermatocytic tumors, epidermoid cysts, neuroendocrine tumors, Leydig cell tumors, Sertoli cell tumors and hemangiomas). Because the proportion of false-positive TGCT was low (5.1%), TGCT not confirmed due to missing pathology report (7.8%, *N* = 43) were included as cases in our analyses.

### Study population

Overall, 1463 eligible subjects have been invited to participate in the study, among which 1367 (93.4%) agreed to participate: 550 TGCT cases, 447 group A and 370 group B controls. Among the total 96 subjects that refused to participate, the most frequent reasons for non-participation were lack of interest in the study (*n* = 32; 33%), no wish to participate (*n* = 27; 28%), or lack of availability (*n* = 20; 21%) (Figure S1). The proportion of men aged 25 years or younger was higher in the non-participants than in the participants (29% versus 11%), while the proportion of men aged 31–35 years old was higher in the participants than in the non-participants (33% versus 21%). In the non-participating men, the proportion of employment in intermediate occupations was higher and the proportion of “not professionally active” was lower than among the eligible subjects that agreed to participate in the study (23% versus 11, and 18% versus 56%, respectively, data not shown). Among the 1367 enrolled men, 853 (62%) agreed to contact their mothers and finally 640 mothers/relatives agreed to participate. Of the subjects that agreed to participate, 44 participants were excluded for not meeting inclusion criteria: *N* = 25 cases were not confirmed by pathology reports (*N* = 21 non GCNIS-related TGCT; *N* = 4 with absence of a tumor); *N* = 5 confirmed GCNIS-related TGCT with time from diagnosis to study inclusion > 12 months (*N* = 4) or with missing date of diagnosis (*N* = 1); *N* = 1 group B control not born in Metropolitan France; and *N* = 13 controls (8 group A and 5 group B) who reported personal history of cryptorchidism. Among eligible subjects (*N* = 1323), 168 did not complete the telephone interview and were excluded (*N* = 48 TGCT cases, *N* = 46 group A controls and *N* = 74 group B controls); reasons for this included refusal (*N* = 44), unreachable subjects after three telephone calls (*N* = 123) and 1 person who passed away prior to interview (Figure S1).

A total of 1124 participants completed the interview, as well as 31 participants’ mothers for whom their son was not interviewed (*N* = 1155). The study population was composed of participants for whom mothers/relatives had completed the interview, and finally included 570 participants’ mothers and 8 participants’ relatives (*N* = 578, 50% participation): 304 TGCT cases − 144 SE (47%) and 132 NS (43%)–, 145 group A controls and 129 group B controls.

### Exposure assessment

Domestic use of pesticides at any time from 1 year before start of pregnancy to 1 year after the son’s birth was collected during the interview with participants’ mothers/relatives through 22 structured questions. The use of insecticides, herbicides and fungicides was assessed for gardening activities, treatment of indoor plants, pest control (against flying insects, crawling insects or termites), treatment of pets, for woodwork and against molds, as well as practice of each activity. Interviewers could provide definition for all items upon request of the interviewee. Frequency of pesticide use was further recorded for pest control (never; less than once a month; less than once a week; more than once a week; daily). Direct use (by the mother/relative) or indirect use (by another person sharing the household, the mother’s spouse for instance) of pesticides was specified for gardening activities and treatment of indoor plants. Furthermore, we computed dichotomous exposure variables related to indoor use of pesticides (ever/never) which comprised treatment of indoor plants, pest controls, treatment of pets, woodwork and molds, and outdoor uses of pesticides (ever/never) that were based on gardening activities and split according to direct or indirect use.

### Statistical analysis

Participants’ characteristics were described by case-control status using mean and SD for continuous variables and frequency and percentage for categorical variables. Odds ratios (OR) and corresponding 95% confidence intervals (CIs) for TGCT in adulthood were estimated for domestic use of pesticides during early periods of development (never use as reference category), using conditional logistic regression models [25]. All models were conditioned for matching factors (hospital center and birth year grouped in 5-year categories) [26]. Trend tests were performed for the ordinal variables reporting frequency of pesticides use. Covariates suspected to be early life risk factors for TGCT were first identified from the literature [27] and considered for adjustment: maternal exposure to diethylstilbestrol during pregnancy (yes, no); maternal smoking during pregnancy (yes, no); breastfeeding (yes, no); personal history of inguinal hernia (yes, no); birth weight (< 2500, 2500–3999, ≥3999 g); gestational age (≤36, > 36 weeks of pregnancy); birth order (first, second, third, fourth and more); sibship size (one, two, three, four and more brothers and sisters); multiple birth (yes, no); family history of testicular cancer (yes, no); family history of cryptorchidism (yes, no); geographic origin (French by birth, by acquisition). In addition, parental occupations at birth and presence of crops in the vicinity of the birthplace were assessed. Parental jobs and industries were coded by a hygienist according to ISCO-1968 (International Standard Classification of Occupations) and NAF-1999 (*Nomenclature d’activités française*). Presence of crops – i.e. arable lands, vineyards, and orchards – in a 500 m buffer around the birthplace (yes, no) was assessed using a geographic information system (GIS) and automatic processing of historical aerial images integrated into the Gouramic software [28]. Each previous suspected covariate was then sequentially added to the model including the exposure variable, and change in OR comparing models with and without the additional covariate was then computed; covariates were retained if the change in OR was > 10%. As none of the covariates assessed led to a change in OR > 10% (Table S1), results presented were computed from models not adjusted for those covariates. As matching on birth year grouped in 5-year intervals resulted in large strata, additional adjustments were performed on age (at diagnosis for cases and inclusion for controls) as a continuous variable to avoid residual confounding by age within the birth year groups.

We estimated OR for TGCT and 95% confidence intervals (CI) according to the histological subtype of the tumors (seminoma and non-seminoma) and tested the heterogeneity of associations using polytomous logistic regression for matched case-control studies (SAS macro *%subtype*) [29]. *P*-values for heterogeneity were derived from the likelihood ratio test [29]. Histological subtype was missing for *N* = 27 cases.

We conducted stratified analysis according to birth cohorts (1970–1980, 1981–1990, and 1991–1999) due to changes in pesticide usage and sales over time. Models stratified by birth cohorts were not conditioned for birth year to avoid over-fitting. Because women living in rural areas, i.e. in the vicinity of agricultural activities, might be exposed to pesticides at higher levels than women from urban areas [30], models were stratified according to the urban or rural status of the birthplace. The urban/rural status of the birthplace was determined according to the population size of the town: birthplaces with less than 2000 inhabitants were considered as rural, whereas the others were urban [31]. Analyses were further stratified on the season during which first trimester of pregnancy occurred (spring/summer, autumn/winter), as prevalence of pesticide use is higher during spring and summer seasons [19, 32]. Finally, the association with the use of pesticides in gardening by the mother/relative and/or another person from the household was stratified by the reported gardening activities of the mother/relative (yes, no) to consider potential exposure due to re-entry tasks during gardening [33, 34]. Effect modifications between domestic use of pesticides and each of the above mentioned strata variables were tested with likelihood ratio tests comparing models with and without interaction terms [25].

The following sensitivity analyses were performed. First, we investigated the effect of domestic use of pesticides on TGCT excluding cases with personal history of cryptorchidism (*N* = 19), and in a second time cases and controls who reported family history of cryptorchidism (*N* = 26) and testicular cancer (*N* = 36). Third, we excluded the TGCT cases not confirmed by pathology reports (*N* = 27) and models were run in this subgroup. We further excluded the participants for which a close relative and not the mother (*n* = 8) answered the interview questions in order to minimize misclassification of exposures during pregnancy.

Domestic exposures to pesticides were only reported if there were at least five exposed cases or controls in exposure categories. For cell counts of five or less, we aggregated cell counts across categories when possible. If the number of exposed cases or controls was less than five and categories could not be combined, risk estimates were not reported because of statistical uncertainty of estimates.

All *P*-values were two-sided and the significance level was set at 0.05. SAS statistical software version 9.4 (SAS Institute Inc., Cary, NC, USA) was used for data analysis.

## Results

The main characteristics of TGCT cases and group A and group B controls are shown in Table 1. Average maternal age at birth was 27.6, 27.0 and 27.8 years, respectively. Low birthweight (< 2500 g) was reported for 6.3% of cases and 5.5%/4.4% of group A and group B controls respectively. The majority of participants were first born (40.5 to 48.1%), and few cases and controls were born from a multiple pregnancy (1.4 to 3.3%). There was no difference between TGCT cases and controls in prenatal and perinatal factors, in parental occupations and in birthplace characteristics. For predisposing factors, family history of testicular cancer was more frequent among TGCT cases than controls (8.2% versus 3.4%/4.7%). There was no difference between the two groups of A and B controls, except for paternal job at birth. Age distribution at diagnosis/ inclusion was slightly different between cases and controls, as there were more cases than controls aged less than 25 years old and more controls than cases aged 31 to 35 years old (Table 2). Among TGCT cases, 43.4% were NS and 47.4% were SE. Age at diagnosis peaked at 26–30 years old for NS and 31–35 years old for SE.

**Table 1:** Characteristics of TGCT cases and controls (group A and group B), *N* = 578, case-control study, France, 2015–2018

	TGCT cases (*N* = 304)	Group A controls (*N* = 145)	Group B controls (*N* = 129)
	n (%)	n (%)	n (%)
**Prenatal Characteristics**			
**Maternal use of diethylstilbestrol during pregnancy**			
No	296 (97.4)	142 (97.9)	129 (100.0)
Yes	1 (0.3)	0 (0.0)	0 (0.0)
Missing	7 (2.3)	3 (2.1)	0 (0.0)
**Maternal smoking during pregnancy**			
No	252 (82.9)	122 (84.1)	110 (85.3)
Yes	52 (17.1)	23 (15.9)	19 (14.7)
**Maternal age (years), mean ± SD**	27.6 ± 4.7	27.0 ± 4.7	27.8 ± 4.6
**Paternal age (years), mean ± SD**	29.8 ± 4.8	29.5 ± 5.3	30.2 ± 5.4
Missing	169 (55.6)	80 (55.2)	62 (48.1)
**Perinatal Characteristics**			
**Inguinal hernia**			
No	263 (86.5)	128 (88.3)	117 (90.7)
Yes	23 (7.6)	8 (5.5)	7 (5.4)
Missing	18 (5.9)	9 (6.2)	5 (3.9)
**Birth weight**			
< 2500 g	19 (6.3)	8 (5.5)	6 (4.7)
[2500–4000[g	250 (82.2)	122 (84.1)	106 (82.2)
> =4000 g	30 (9.9)	15 (10.3)	16 (12.4)
Missing	5 (1.6)	0 (0.0)	1 (0.8)
**Gestational age**			
< =36 weeks	27 (8.9)	10 (6.9)	8 (6.2)
> 36 weeks	270 (88.8)	134 (92.4)	120 (93.0)
Missing	7 (2.3)	1 (0.7)	1 (0.8)
**Birth order**			
First	123 (40.5)	61 (42.1)	62 (48.1)
Second	101 (33.2)	49 (33.8)	41 (31.8)
Third	49 (16.1)	23 (15.9)	15 (11.6)
Fourth and more	31 (10.2)	12 (8.3)	11 (8.5)
**Sibship size**			
1	21 (6.9)	23 (15.9)	14 (10.9)
2	148 (48.7)	48 (33.1)	55 (42.6)
3	96 (31.6)	48 (33.1)	40 (31.0)
> =4	39 (12.8)	26 (17.9)	20 (15.5)
**Birth from multiple pregnancy**			
No	276 (90.8)	134 (92.4)	122 (94.6)
Yes	10 (3.3)	2 (1.4)	3 (2.3)
Missing	18 (5.9)	9 (6.2)	4 (3.1)
**Birth cohort**			
1970–1980	89 (29.3)	47 (32.4)	34 (26.4)
1981–1990	162 (53.3)	88 (60.7)	88 (68.2)
1991–2000	53 (17.4)	10 (6.9)	7 (5.4)
**Predisposing Characteristics**			
**Geographic origin**			
French by birth	284 (93.4)	136 (93.8)	123 (95.3)
French by acquisition	2 (0.7)	0 (0.0)	2 (1.6)
Missing	18 (5.9)	9 (6.2)	4 (3.1)
**Family history of TGCT**			
No	260 (85.5)	131 (90.3)	119 (92.2)
Yes	25 (8.2)	5 (3.4)	6 (4.7)
Missing	19 (6.3)	9 (6.2)	4 (3.1)
**Family history of cryptorchidism**			
No	264 (86.8)	131 (90.3)	122 (94.6)
Yes	19 (6.3)	4 (2.8)	3 (2.3)
Missing	21 (6.9)	10 (6.9)	4 (3.1)
**Parental Socio-Economic Status**			
**Maternal job at birth (ISCO-1968 codes)**			
Not working at time of birth	111 (36.5)	61 (42.1)	48 (37.2)
Professional, Technical and Related Workers (0/1)	57 (18.8)	28 (19.3)	34 (26.4)
Administrative and Managerial Workers (2)	9 (3.0)	0 (0.0)	5 (3.9)
Clerical and Related Workers (3)	61 (20.1)	26 (17.9)	23 (17.8)
Sales Workers (4)	14 (4.6)	1 (0.7)	5 (3.9)
Service Workers (5)	27 (8.9)	14 (9.7)	10 (7.8)
Agricultural, Animal Husbandry and Forestry Workers, Fishermen and Hunters (6)	8 (2.6)	4 (2.8)	1 (0.8)
Production and Related Workers, Transport Equipment Operators and Labourers (7/8/9)	17 (5.6)	11 (7.6)	3 (2.3)
**Maternal job at birth’s industry (NAF-1999 codes)**			
Not working at time of birth	111 (36.5)	61 (42.1)	48 (37.2)
Agriculture,hunting and forestry (A)	11 (3.6)	4 (2.8)	1 (0.8)
Mining and quarrying (C)	0 (0.0)	1 (0.7)	0 (0.0)
Manufacturing (D)	27 (8.9)	15 (10.3)	10 (7.8)
Electricity,gas and water supply (E)	0 (0.0)	1 (0.7)	0 (0.0)
Construction (F)	2 (0.7)	0 (0.0)	1 (0.8)
Wholesale and retail trade;repair of motor vehicles,motorcycles and personal and household goods (G)	16 (5.3)	4 (2.8)	10 (7.8)
Hotels and restaurants (H)	3 (1.0)	3 (2.1)	1 (0.8)
Transport,storage and communication (I)	10 (3.3)	3 (2.1)	6 (4.7)
Financial intermediation (J)	12 (3.9)	4 (2.8)	6 (4.7)
Real estate,renting and business activities (K)	12 (3.9)	2 (1.4)	5 (3.9)
Public administration and defence;compulsory social security (L)	14 (4.6)	12 (8.3)	4 (3.1)
Education (M)	30 (9.9)	11 (7.6)	14 (10.9)
Health and social work (N)	46 (15.1)	20 (13.8)	21 (16.3)
Other community,social and personal service acitivities (O)	10 (3.3)	1 (0.7)	2 (1.6)
Private houeholds with employed persons (P)	0 (0.0)	3 (2.1)	0 (0.0)
**Paternal job at birth (ISCO-1968 codes)**			
Not working at time of birth	42 (13.8)	20 (13.8)	11 (8.5)
Professional, Technical and Related Workers (0/1)	65 (21.4)	39 (26.9)	51 (39.5)
Administrative and Managerial Workers (2)	24 (7.9)	4 (2.8)	12 (9.3)
Clerical and Related Workers (3)	24 (7.9)	14 (9.7)	12 (9.3)
Sales Workers (4)	13 (4.3)	6 (4.1)	8 (6.2)
Service Workers (5)	24 (7.9)	5 (3.4)	6 (4.7)
Agricultural, Animal Husbandry and Forestry Workers, Fishermen and Hunters (6)	21 (6.9)	11 (7.6)	2 (1.6)
Production and Related Workers, Transport Equipment Operators and Labourers (7/8/9)	89 (29.3)	46 (31.7)	27 (20.9)
Military	2 (0.7)	0 (0.0)	0 (0.0)
**Paternal job at birth’s industry (NAF-1999 codes)**			
Not working at time of birth	42 (13.8)	20 (13.8)	11 (8.5)
Agriculture,hunting and forestry (A)	25 (8.2)	11 (7.6)	3 (2.3)
Manufacturing (D)	55 (18.1)	30 (20.7)	25 (19.4)
Electricity,gas and water supply (E)	3 (1.0)	1 (0.7)	3 (2.3)
Construction (F)	28 (9.2)	12 (8.3)	10 (7.8)
Wholesale and retail trade;repair of motor vehicles,motorcycles and personal and household goods (G)	21 (6.9)	10 (6.9)	12 (9.3)
Hotels and restaurants (H)	7 (2.3)	3 (2.1)	2 (1.6)
Transport,storage and communication (I)	28 (9.2)	15 (10.3)	12 (9.3)
Financial intermediation (J)	12 (3.9)	3 (2.1)	5 (3.9)
Real estate,renting and business activities (K)	21 (6.9)	10 (6.9)	11 (8.5)
Public administration and defence;compulsory social security (L)	30 (9.9)	12 (8.3)	9 (7.0)
Education (M)	16 (5.3)	12 (8.3)	12 (9.3)
Health and social work (N)	13 (4.3)	4 (2.8)	9 (7.0)
Other community,social and personal service acitivities (O)	3 (1.0)	2 (1.4)	4 (3.1)
Extra-territorial organizations and bodies (Q)	0 (0.0)	0 (0.0)	1 (0.8)
**Maternal education**			
Baccalaureate’s degree or less	192 (63.2)	83 (57.2)	59 (45.7)
Graduate studies	84 (27.6)	35 (24.1)	52 (40.3)
Other	28 (9.2)	26 (17.9)	18 (14.0)
Missing	0 (0.0)	1 (0.7)	0 (0.0)
**Parental incomes at birth**			
0 to less than 5000€	63 (20.7)	42 (29.0)	35 (27.1)
5000 to less than 10,000€	55 (18.1)	22 (15.2)	18 (14.0)
10,000 to less than 20,000€	41 (13.5)	22 (15.2)	16 (12.4)
20,000 to less than 30,000€	21 (6.9)	5 (3.4)	13 (10.1)
30,000€ and more	24 (7.9)	5 (3.4)	5 (3.9)
Missing	100 (32.9)	49 (33.8)	42 (32.6)
**Residential Characteristics**			
**Urban status of the place of birth,commune level**			
No	73 (24.0)	36 (24.8)	27 (20.9)
Yes	231 (76.0)	109 (75.2)	102 (79.1)
**Presence of crops in 500 m buffer around place of birth**			
No	76 (25.0)	39 (26.9)	38 (29.5)
Yes	223 (73.4)	104 (71.7)	90 (69.8)
Missing	5 (1.6)	2 (1.4)	1 (0.8)
**Possession of a garden**			
No	153 (50.3)	76 (52.4)	64 (49.6)
Yes	151 (49.7)	69 (47.6)	65 (50.4)
**Possession of a vegetable garden**			
No	223 (73.4)	97 (66.9)	88 (68.2)
Yes	81 (26.6)	48 (33.1)	41 (31.8)
**Possession of an orchard**			
No	261 (85.9)	127 (87.6)	110 (85.3)
Yes	43 (14.1)	18 (12.4)	19 (14.7)
**Gardening activities during the spring/summer seasons**			
No	230 (75.7)	102 (70.3)	92 (71.3)
Yes	74 (24.3)	42 (29.0)	37 (28.7)
Missing	0 (0.0)	1 (0.7)	0 (0.0)
**Possession of pets**			
No	69 (22.7)	32 (22.1)	29 (22.5)
Yes	235 (77.3)	113 (77.9)	100 (77.5)
**Possession of indoor plants**			
No	178 (58.6)	81 (55.9)	78 (60.5)
Yes	126 (41.4)	64 (44.1)	51 (39.5)

**Table 2:** Distribution of TGCT cases, overall and by subtypes, and group A and group B controls by age at diagnosis/inclusion (years), case-control study, *N* = 578, France, 2015–2018

Age at diagnosis/inclusion (years)	Group A controls	Group B controls	TGCT cases^*a*^	Non-seminoma cases	Seminoma cases
	n (%)	n (%)	n (%)	n (%)	n (%)
<=25	7 (4.8)	5 (3.9)	54 (17.8)	35 (26.5)	13 (9.0)
26–30	23 (15.9)	28 (21.7)	79 (26.0)	38 (28.8)	32 (22.2)
31–35	63 (43.4)	55 (42.6)	84 (27.6)	30 (22.7)	44 (30.6)
36–40	36 (24.8)	32 (24.8)	59 (19.4)	20 (15.2)	38 (26.4)
> = 41	16 (11.0)	9 (7.0)	28 (9.2)	9 (6.8)	17 (11.8)
Total	145 (100.0)	129 (100.0)	304 (100.0)	132 (100.0)	144 (100.0)

^*a*^ Include *N* = 1 confirmed TGCT case of regressed germ cell tumours (GCNIS-related TGCT, unknown subtype) and *N* = 27 TGCT cases not confirmed by pathology report. As the median delay between date of diagnosis and date of inclusion among the cases confirmed by pathology report was 8 days, we used age at inclusion instead of age at diagnosis for the 27 cases not confirmed by pathology report

A comparison of subjects’ characteristics of mothers/relatives interviewed (*N* = 578), and of mothers not interviewed, did not show differences in terms of participants’ pre- and perinatal factors or socio-economic status at interview, nor differences of mothers’ socio-economic status at subjects’ birth. Family history of TGCT was more frequent in sons of mothers interviewed. As the participation rate differed between case mothers (52.6%) and control mothers (40.1%), there was a higher proportion of cases in the group of mothers interviewed and age distribution of cases and controls was different between the two groups (Table S2). Prevalence of domestic use of pesticides from 1 year before start of pregnancy to 1 year after birth in the study population (N = 578) was 77.3% for insecticides, 15.9% for fungicides, 12.1% for herbicides and 78.9% for pesticides overall (Table 3, Table S3). As group A and group B controls were not significantly different based on socio-demographic, pre-, perinatal and predisposing characteristics, there were grouped in the analyses. There was no association between TGCT risk and domestic use of insecticides (OR = 1.27, CI = 0.80–2.01), herbicides (OR = 1.15, CI = 0.67–2.00), as well as pesticides overall (OR = 1.18, CI = 0.74–1.88) (Table 3, Table S3). We observed statistically significant increased TGCT risk (OR = 1.73, CI = 1.04–2.87) and NS risk (OR = 2.44, CI = 1.26–4.74) for the domestic use of fungicides, while no association was found for SE. Adjustment on age for main exposures and outcomes showed slightly lower associations as observed in the main analysis (Table S4). The observed association between domestic use of fungicides and TGCT risk provided similar OR but was no longer statistically significant (OR = 1.55, CI = 0.91–2.65). However, the association observed for domestic use of fungicides and risk of NS remained unchanged (OR = 2.40, CI = 1.17–4.91) (Table S4).

**Table 3:** Odds ratios and 95% confidence intervals for TGCT associated with domestic use of pesticides during early periods of development, overall and according to histological subtypes, case-control study, *N* = 578, France, 2015–2018

	Total	Controls A + B	All TGCT cases		Non-Seminomas		Seminomas		P-HET^*b*^
	(*N* = 578)	(*N* = 274)	(*N* = 304)		(*N* = 132)		(*N* = 144)		
	n (%)	n (%)	n (%)	OR^*a*^ (95% CI)	n (%)	OR^*a*^ (95% CI)	n (%)	OR^*a*^ (95% CI)	
**Domestic use of insecticides**									0.12
No	129 (22.3)	62 (22.6)	67 (22)	1.00	28 (21.2)	1.00	34 (23.6)	1.00	
Yes	447 (77.3)	211 (77.0)	236 (77.6)	1.27 (0.80, 2.01)	104 (78.8)	1.78 (0.91, 3.48)	109 (75.7)	0.89 (0.50, 1.59)	
Missing	2 (0.3)	1 (0.4)	1 (0.3)	–	0 (0.0)	–	1 (0.7)	–	
**Domestic use of fungicides**									0.08
No	478 (82.7)	234 (85.4)	244 (80.3)	1.00	100 (75.8)	1.00	123 (85.4)	1.00	
Yes	92 (15.9)	37 (13.5)	55 (18.1)	1.73 (1.04, 2.87)	31 (23.5)	2.44 (1.26, 4.74)	18 (12.5)	1.04 (0.52, 2.07)	
Missing	8 (1.4)	3 (1.1)	5 (1.6)	–	1 (0.8)	–	3 (2.1)	–	
**Domestic use of herbicides**									0.17
No	508 (87.9)	242 (88.3)	266 (87.5)	1.00	111 (84.1)	1.00	129 (89.6)	1.00	
Yes	70 (12.1)	32 (11.7)	38 (12.5)	1.15 (0.67, 2.00)	21 (15.9)	1.64 (0.83, 3.27)	15 (10.4)	0.80 (0.37, 1.71)	
Missing	0 (0.0)	0 (0)	0 (0.0)	–	0 (0.0)	–	0 (0.0)	–	
**Domestic use of any pesticides**									0.18
No	119 (20.6)	57 (20.8)	62 (20.4)	1.00	25 (18.9)	1.00	32 (22.2)	1.00	
Yes	456 (78.9)	215 (78.5)	241 (79.3)	1.18 (0.74, 1.88)	107 (81.1)	1.56 (0.79, 3.06)	111 (77.1)	0.84 (0.47, 1.51)	
Missing	3 (0.5)	2 (0.7	1 (0.3)	–	0 (0.0)	–	1 (0.7)	–	

*OR* odds ratios, *CI* confidence intervals

^*a*^ Estimates obtained comparing TGCT cases to group A and group B controls combined. Analysis was restricted to subjects with no missing data for the exposure variable (0.2 to 1.4% excluded)

^*b*^ *P*-value for heterogeneity derived from the Likelihood Ratio Test, comparing seminoma versus non-seminoma tumours

Tables 4 and S5 present the ORs for domestic use of pesticides according to specific household applications, for exposure categories with at least 5 cases and 5 controls. No association was found for the domestic use of pesticides during gardening activities, for treatment of indoor plants, and for pest control. We observed a statistically significant increased OR for NS associated with the use of fungicides and/or insecticides for woodwork (OR = 2.35, CI = 1.06–5.20) with heterogeneity in this association for NS versus SE (p-for-heterogeneity = 0.03). The use of insecticides on cats and dogs was associated with a statistically significant increase in NS risk (OR = 1.95, CI = 1.12–3.40) and was found different from the association with SE (p-for-heterogeneity = 0.01). No significant trends were noticed with insecticides use frequency against crawling or flying insects respectively (Table 4). Indoor use and direct outdoor use of pesticides were not associated with TGCT risk (OR = 1.14, CI = 0.72–1.79 and OR = 0.74, CI = 0.39–1.39, respectively), while a statistically significant increase in NS risk was observed for indirect outdoor use of pesticides (OR = 2.33, CI = 1.24–4.39) (Table S6).

**Table 4:** Odds ratios and 95% confidence intervals for TGCT associated with domestic use of pesticides during early periods of development, by household applications, overall and according to histological subtypes, case-control study, *N* = 578, France, 2015–2018

	Total	Controls A + B	All TGCT cases		Non-Seminomas		Seminomas		P-HET^*b*^
	(*N* = 578)	(*N* = 274)	(*N* = 304)		(*N* = 132)		(*N* = 144)		
	n (%)	n (%)	n (%)	OR^*a*^ (95% CI)	n (%)	OR^*a*^ (95% CI)	n (%)	OR^*a*^ (95% CI)	
**For Gardening**									
**Use of pesticides**									0.08
No	433 (74.9)	206 (75.2)	227 (74.7)	1.00	90 (68.2)	1.00	116 (80.6)	1.00	
Yes	138 (23.9)	65 (23.7)	73 (24.0)	1.18 (0.77, 1.82)	39 (29.5)	1.60 (0.92, 2.78)	27 (18.8)	0.78 (0.43, 1.40)	
Missing	7 (1.2)	3 (1.1)	4 (1.3)	–	3 (2.3)	–	1 (0.7)	–	
**Use of insecticides**									0.17
No	509 (88.1)	240 (87.6)	269 (88.5)	1.00	114 (86.4)	1.00	130 (90.3)	1.00	
Yes	69 (11.9)	34 (12.4)	35 (11.5)	1.11 (0.63, 1.94)	18 (13.6)	1.66 (0.83, 3.33)	14 (9.7)	0.79 (0.36, 1.73)	
Missing	0 (0.0)	0 (0.0)	0 (0.0)	–	0 (0.0)	–	0 (0.0)	–	
**Use of herbicides**									0.17
No	508 (87.9)	242 (88.3)	266 (87.5)	1.00	111 (84.1)	1.00	129 (89.6)	1.00	
Yes	70 (12.1)	32 (11.7)	38 (12.5)	1.15 (0.67, 2.00)	21 (15.9)	1.64 (0.83, 3.27)	15 (10.4)	0.80 (0.37, 1.71)	
Missing	0 (0.0)	0 (0.0)	0 (0.0)	–	0 (0.0)	–	0 (0.0)	–	
**Use of fungicides**									0.75
No	541 (93.6)	257 (93.8)	284 (93.4)	1.00	122 (92.4)	1.00	135 (93.8)	1.00	
Yes	37 (6.4)	17 (6.2)	20 (6.6)	1.22 (0.59, 2.54)	10 (7.6)	1.28 (0.50, 3.30)	9 (6.3)	1.03 (0.40, 2.65)	
Missing	0 (0.0)	0 (0.0)	0 (0.0)	–	0 (0.0)	–	0 (0.0)	–	
**On Indoor Plants**^**c**^									
**Use of insecticides**									0.82
No	545 (94.3)	262 (95.6)	283 (93.1)	1.00	124 (93.9)	1.00	133 (92.4)	1.00	
Yes	28 (4.8)	10 (3.6)	18 (5.9)	2.06 (0.89, 4.75)	7 (5.3)	1.90 (0.62, 5.80)	10 (6.9)	2.26 (0.83, 6.15)	
Missing	5 (0.9)	2 (0.7)	3 (1.0)	–	1 (0.8)	–	1 (0.7)	–	
**For Pest Control**									
**Use of insecticides**									0.19
No	174 (30.1)	82 (29.9)	92 (30.3)	1.00	38 (28.8)	1.00	45 (31.3)	1.00	
Yes	404 (69.9)	192 (70.1)	212 (69.7)	1.12 (0.75, 1.69)	94 (71.2)	1.49 (0.83, 2.67)	99 (68.8)	0.89 (0.54, 1.49)	
Missing	0 (0.0)	0 (0.0)	0 (0.0)	–	0 (0.0)	–	0 (0.0)	–	
**Insecticide use frequency against crawling insects**									0.58
No use	371 (64.2)	183 (66.8)	188 (61.8)	1.00	79 (59.8)	1.00	93 (64.6)	1.00	
Less than once a month	153 (26.5)	67 (24.5)	86 (28.3)	1.31 (0.85, 2.02)	40 (30.3)	1.65 (0.90, 3.04)	36 (25.0)	1.07 (0.62, 1.86)	
Less than once a week	32 (5.5)	17 (6.2)	15 (4.9)	0.83 (0.38, 1.80)	7 (5.3)	0.98 (0.35, 2.73)	8 (5.6)	0.73 (0.29, 1.88)	
More than once a week to daily	20 (3.5)	6 (1.8)	14 (3.3)	1.78 (0.56, 5.60)	5 (3.8)	1.43 (0.27, 7.68)	7 (4.9)	2.07 (0.48, 9.00)	
Missing	2 (0.3)	1 (0.4)	1 (0.3)	–	1 (0.8)	–	0 (0.0)	–	
***P trend***				*0.41*		*0.39*		*0.80*	
**Insecticide use frequency against flying insects**									0.33
No use	228 (39.4)	106 (38.7)	122 (40.1)	1.00	51 (38.6)	1.00	58 (40.3)	1.00	
Less than once a month	153 (26.5)	73 (26.6)	80 (26.3)	1.16 (0.73, 1.85)	36 (27.3)	1.53 (0.79, 2.96)	40 (27.8)	0.93 (0.52, 1.67)	
Less than once a week	83 (14.4)	40 (14.6)	43 (14.1)	0.94 (0.53, 1.67)	22 (16.7)	1.43 (0.67, 3.05)	16 (11.1)	0.62 (0.28, 1.36)	
More than once a week	77 (13.3)	39 (14.2)	38 (12.5)	0.94 (0.52, 1.70)	16 (12.1)	1.17 (0.50, 2.76)	19 (13.2)	0.87 (0.41, 1.83)	
Daily	35 (6.1)	14 (5.1)	21 (6.9)	1.98 (0.89, 4.44)	7 (5.3)	1.21 (0.36, 4.10)	11 (7.6)	2.60 (0.95, 7.10)	
Missing	2 (0.3)	2 (0.7)	0 (0.0)	–	0 (0.0)	–	0 (0.0)	–	
***P trend***				*0.45*		*0.59*		*0.52*	
**Wood and Mold**^**d**^									
**Use of fungicides and/or insecticides for woodwork**									0.03
No	517 (89.4)	249 (90.9)	268 (88.2)	1.00	110 (83.3)	1.00	134 (93.1)	1.00	
Yes	57 (9.9)	24 (8.8)	33 (10.9)	1.36 (0.73, 2.55)	21 (15.9)	2.35 (1.06, 5.20)	8 (5.6)	0.58 (0.22, 1.53)	
Missing	4 (0.7)	1 (0.4)	3 (1.0)	–	1 (0.8)	–	2 (1.4)	–	
**Pets**									
**Use of insecticides on cats and dogs**									0.01
No	410 (70.9)	195 (71.2)	215 (70.7)	1.00	85 (64.4)	1.00	114 (79.2)	1.00	
Yes	163 (28.2)	76 (27.7)	87 (28.6)	1.14 (0.76, 1.71)	45 (34.1)	1.95 (1.12, 3.40)	30 (20.8)	0.66 (0.38, 1.15)	
Missing	5 (0.9)	3 (1.1)	2 (0.7)	–	2 (1.5)	–	0 (0.0)	–	
**Use of insecticides on other animals**^**e**^									
No	565 (97.8)	268 (97.8)	297 (97.7)	1.00					
Yes	11 (1.9)	6 (2.2)	5 (1.6)	0.38 (0.09, 1.61)					
Missing	2 (0.3)	0 (0.0)	2 (0.7)	–					

*OR* odds ratios, *CI* confidence intervals

^*a*^ Estimates obtained comparing TGCT cases to group A and group B controls combined. Analysis was restricted to subjects with no missing data for the exposure variable (0.2 to 1.4% excluded)

^*b*^ *P*-value for heterogeneity derived from the Likelihood Ratio Test, comparing seminoma versus non-seminoma tumours

^c^ Use of fungicides on indoors plants was not presented because some cells had less than 5 subjects

^d^ Use of fungicides on mold was not presented because some cells had less than 5 subjects

^e^ Analysis by subtype for use of insecticides on other animals was not presented because some cells had less than 5 subjects

There was no effect modification by birth cohorts and no association was observed overall (Table S7). We found no effect modification by status of birthplace and no association overall (Table S8). Neither effect modification nor associations were observed when stratifying on the season during which the first trimester of pregnancy occurred (Table S9). Among mothers/relatives who reported doing gardening activities, the use of pesticides in gardening by another person sharing the household was 30.1% (*N* = 46/153), while it was 9.7% (*N* = 41/424) among those who did not (Table S10). Although there was no effect modification by reported gardening activities (p-for-interaction = 0.63), the use of pesticides during gardening by another person sharing the household was positively associated with TGCT and NS in particular among mothers/relatives who reported not doing gardening activities (OR = 2.30, CI = 1.06–4.96, and OR = 4.75, CI = 1.83–12.34, respectively); there was no association among mothers/relatives who reported doing gardening activities.

Excluding TGCT cases with personal history of cryptorchidism did not modify our results; a positive OR was observed for the domestic use of fungicides (OR = 1.78, CI = 1.06–2.99) (Table S11). In the subpopulation restricted to participants with no family history of cryptorchidism and no family history of testicular cancer, our results were similar to the main results. We observed statistically significant positive associations with TGCT for the domestic use of fungicides (OR = 1.92, CI = 1.12–3.30) (Table S11). Excluding TGCT cases not confirmed by pathology reports did not change our results (Table S11). The associations with domestic use of fungicides remained statistically significant, with OR for TGCT of 1.68 (CI = 1.00–2.83). Restricting the analyses to participants with interviewed mothers only did not change the observed associations with domestic use of fungicides (OR = 1.82, CI = 1.09–3.03) (Table S11).

## Discussion

In this French case-control study, domestic use of fungicides during early periods of development was found positively associated with increased risk of TGCT in the sons, driven by an association with the histological subtype NS, while no association was found for domestic use of insecticides and herbicides. Additional adjustment for age at diagnosis/inclusion did not alter the main conclusion. When focusing on the different household applications, the results also suggest positive associations between risk of NS and use of insecticides on pets and use of fungicides and/or insecticides for woodwork.

Prevalence of domestic use of pesticides was high in the study population and was driven by the use of insecticides (77%); the prevalence of use of fungicides was much lower (16%). Prevalence was similar to that of other studies conducted in France, notably for the observed higher prevalence of insecticide use and much lower prevalence of fungicide use [11, 32, 35, 36], and worldwide [37–39]; although published data covered more recent time periods.

To our knowledge, the only published data on specific pesticides used domestically during the study period are available from the USA [40, 41], Canada [42] and UK [43]. Based on these studies, even if active ingredients reported were not always the same, organophosphorus, pyrethroids, carbamates and acid herbicides pesticides appeared to be most commonly used during the period of interest. Regarding fungicides, the American National Household Pesticide Usage Study (1976–1977) [41] reported that Captan (phthalimide) and Folpet (dicarboximide) were among the most used pesticides in households during this period. These families were still in use in France in 2010, according to The French Agency for Food, Environmental and Occupational Health & Safety [44]. Organochlorine insecticides, however, disappeared at the turn of the 90’s; and the use of pyrethroids compounds seemed to increase as the use of organophosphorus compounds was decreasing.

No previous study found increased risks of TGCT and NS for the use of fungicides; the main reason being however that fungicides were not specifically investigated in other studies. In contrast to our results, an increased risk of TGCT was observed for self-reported household use of insecticides (OR = 3.23, CI = 1.15–9.11) [13]. In addition, positive associations have been found for gardening tasks, with OR for TGCT of 1.84 (CI = 1.23–2.75) in a French case-control study [15], and for the use of pesticides in the garden, with OR for TGCT from 1.83 (CI 1. 02–3.29) to 4.80 (CI = 0.91–25.30) and OR for NS of 2.54 (CI = 1.26–5.11) in Italian settings [13, 14]. However, these studies covered adulthood exposure periods, and part of these results did not remain statistically significant in multivariate models. Moreover, it is noteworthy that cryptorchidism may be an unnecessary adjustment in these studies in light of the current understanding of the TDS [7]. In our study, controls with personal history of cryptorchidism were not eligible. Moreover, because cases and controls with personal and/or family history of cryptorchidism and testicular cancer may be at higher risk of developing TGCT, they were excluded in sensitivity analyses, and the previously observed associations remained.

There was no heterogeneity between birth cohorts and urban/rural status of birthplace. Results were obtained with a large uncertainty because of small numbers and we cannot exclude that they were due to chance. No clear findings have been reported on the association between living in a rural environment and risk of TGCT; heterogeneity in the definition of rural areas might be a reason [8, 14, 15].

Our analyses showed a positive association for the risk of NS with the use of pesticides for gardening by another person than the mother/relative sharing the same household (most likely to be the cases’/controls’ father although this has not been investigated), and this was particularly observed among mothers who had not reported any gardening activities. While this observation might suggest a possible role of indirect maternal exposures, it might also support the hypothesis of a potential role of paternal exposures during early periods of development. Exposures to endocrine disrupting chemicals (EDCs), especially during preconception, have been suggested to cause epigenetic changes in the male germline, which can persist and alter embryonic development and further affect birth outcomes, the reproductive system and genital development [17]. However, this result might also be due to chance and more research is needed to confirm the role of paternal exposures during preconception.

Looking at early periods of development, parental occupational pesticide exposure before birth was not found associated with risk of TGCT in the offspring in a large register-based case-control study conducted in the Nordic countries, although the job-exposure matrix used only considered farmers as exposed to pesticides and there was no detail on the type of pesticides (insecticides, herbicides, fungicides) [45]. In Denmark, however, an increased OR for TGCT was observed for paternal wood-related occupations with potential exposure to wood preservatives that contain insecticides and fungicides (OR = 1.50, CI = 1.01–2.24) [46]. Furthermore, parental domestic exposure to pesticides during pregnancy has been found positively associated with hypospadias [47] and cryptorchidism [48]; however results are currently not conclusive. Although we intended to cover early periods of development, we were unable to clearly distinguish between preconception, pregnancy, birth and early infancy exposures, and in particular the first trimester of pregnancy with respect to testicular development [19]. In an attempt to focus on this specific window, we were able to stratify on the season during which the first trimester of pregnancy occurred, and no statistically significant difference in the risk of TGCT was observed between summer/spring and autumn/winter.

Our findings showed stronger associations for NS than SE; overall, no association was found for SE, regardless of household applications and types of pesticides. Given the earlier onset of NS compared to SE (median age at diagnosis of 25 versus 35 years), it has been hypothesized that perinatal factors may be more relevant for NS than SE [49]. However, the available literature rather reports that SE and NS share important etiologic factors [50, 51]. Also, due to the low numbers and large uncertainty for some of our risk estimates, other studies are warranted to confirm these observations.

The presence of GCNIS cells and increasing understanding of molecular mechanisms support the hypothesis of a developmental origin of TGCT with disruption of primordial germ cell differentiation. Exposure to EDCs has been suggested to cause intrauterine hormonal imbalance through impairing androgen signalling or mimicking oestrogen signalling, leading to alteration of foetal gonadal development and negative health outcomes in the offspring [5, 9, 52]. In the testes, experimental studies have shown that fungicides can inhibit testicular cytochrome P450 activity – responsible for the detoxication and bioactivation of drugs, carcinogens and pesticides –, induce oxidative damages in the testes leading to apoptosis as well as anti-androgenic effects in male rats [53–55]. Transgenerational epimutations of primordial germ cells were observed following exposure to vinclozolin fungicide [56]. Insecticide and herbicide compounds have shown similar endocrine disruption activities in the testes [57, 58]. In epidemiological studies, no clear association between elevated maternal serum concentrations of hexachlorobenzene fungicide and TGCT risk has been found so far [59, 60], while increased risks were observed for p,p’-dichlorodiphenyldichloroethylene and chlordane organochlorine insecticides; although serum was collected around son’s diagnosis [59, 61, 62]. In our study population, users of fungicides were also likely users of insecticides and/or herbicides. Therefore, we cannot surely differentiate an independent effect of fungicides compare to insecticides and herbicides on TGCT risk. Moreover, there might be combined or synergetic effects of pesticides compounds, which we were not able to show with our data. A large number of insecticides, herbicides and fungicides compounds acting as EDCs remain to be investigated in relation to TGCT occurrence, and future research should focus on early periods of development and domestically used compounds [9, 63].

To our knowledge, this is the first study investigating the domestic use of pesticides during early periods of development in association with TGCT occurrence in young men. Strengths of the study include the prospective and multicentric recruitment of cases and controls, covering the whole Metropolitan French territory, and the inclusion of participants’ mothers/relatives who provided information specific to preconception, pregnancy and early infancy periods [22]. Interviews were conducted by trained investigators blinded to the case-control status of participants and participants’ mothers, and contained detailed questions on the domestic use of pesticides, covering several types of pesticides and household applications. We were able to estimate TGCT risk according to the subtypes NS and SE. The large number of pre- and perinatal covariates collected, covering most of established and suspected TGCT risk factors, allowed us to assess confounding, and our results were similar with or without adjustment for these covariates. We were able to take into account other sources of pesticide exposure, such as parental occupation at birth and presence of crops around residence at birth, for which adjustment did not change the results.

Our study has several limitations. First, inherent in case-control studies relying on self-reported information, reporting bias (accuracy in general) and recall bias (different reporting by case-control status) is of concern, the latter possibly due to over-reporting of cases or underreporting of controls or their mothers. Notably, participation of mothers was higher among cases than controls (64% versus 40%). Over-reporting among cases or underreporting among controls would both lead to an overestimation of our effect estimates [64]. The use of handouts and structured questionnaires, and telephone interviews blinded to the case-control status were nevertheless intended to minimize recall bias. Moreover, intense recall bias would probably have resulted in generalized increased prevalence among cases and ORs – which was not observed here – and not to the association observed specifically with fungicides. Because participants’ mothers were asked to remember their use of pesticides up to 45 years ago, random reporting error is an issue, possibly leading to an attenuation of any true effect. For very few participants, a close relative was interviewed. As the recall of exposures during pregnancy and the peri-natal period may be less accurate in relatives compared to mothers, a sensitivity analysis restricting the analyses to mothers interviewed was also performed, showing no change in the observed associations. Further, it would have been difficult to accurately collect more detailed information, such as the quantity of pesticides applied or the specific active ingredients used. The retrospective assessment of pesticide uses during critical time windows such as before pregnancy and after birth is challenging, and its validity can be of concern, particularly when the time lag between exposure and reporting is large. Studies have shown moderate to high reproducibility for self-reported past pesticides uses in epidemiological settings, in particular for general pesticide categories (insecticides, herbicides and fungicides), based on repeated interviews or correlations with pesticide concentration in dust. Moreover, reliability has been found similar among cases and controls suggesting that differential recall was likely to be minimal [65–68]. In our study, self-reporting was the only means for retrospectively assessing perinatal domestic use of pesticides – other methods such as biomarkers or measurements of pesticide concentration were not feasible. Our questionnaire collected information for the three major types of pesticides (insecticides, herbicides and fungicides), and the different situations they could be used at home. Frequency of use and user (mother or another person in the household) were also collected for some items. We intended to reduce reporting errors by training of interviewers, and thorough definitions and explanations available for each questionnaire item.

Low response rates are a major challenge to epidemiological research. The characteristics of the target population of the present study (i.e. young adult men) are particularly associated with low response rate in the literature [69, 70], making it difficult to obtain an unbiased random sample of controls from the general population. As testicular cancer and reproduction are sensitive topics, as well as to minimize non-response bias, and increase consent to blood sampling, the study was conducted in the University Hospitals, with similar catchment populations for cases, controls A and controls B, which was the approach that was the most promising one resulting from our pilot study [23]. The high response rates of eligible subjects to whom the study was proposed is similar to previous studies [14, 71, 72]. The non-participants were more likely younger. Also, in the non-participants the proportion of employment in intermediate occupations was higher and the proportion of “not professionally active” was lower than among the subjects that agreed to participate in the study (23% versus 11, and 18% versus 56%, respectively, data not shown). Case/controls status of non-respondents was unfortunately not available. As we cannot exclude in the clinical setting some underreporting of eligible cases and controls not interested in participating, the response rate is likely to be overestimated. Yet, the financial compensation for participation in interview and blood sampling, as well as the reimbursement per enrolled participant to the investigating centers of the staff costs for recruitment, may have facilitated recruitment, and resulted in a higher response rate compared to a previous French hospital-based case-control study on risk factors of TGCT with cases recruited in CECOS for sperm cryopreservation and controls in maternity clinics of the same University hospitals [15]. This study conducted between 2002 and 2005, included 81% of the eligible TGCT cases to whom the study was proposed, and 39% of controls.

Another main limitation of our study is our inability to ascertain whether the controls are representative for the source population from which the cases originate. The ascertainment protocol recruits controls from hospitals where the men attend for particular purposes [22, 23]. Hence, they may differ in relevant criteria from men in the underlying source population not attending the hospital for the same purposes. Whether domestic use of pesticides is among those is rather speculative. Moreover, recruitment of controls was performed in centers with regional scale and activity to avoid over-matching of cases and controls. Also, the choice of fecund controls allowed minimizing the risk of recruiting controls with fertility disorders that may originate in the TDS [7]. To be noted, that the two independently collected groups of controls did not differ in terms of early life characteristics so that they could be combined to increase the sample size. While the response rate of mothers for which the participating sons agreed to give a contact was good (640 of the 853 subjects contacted, i.e. 75%), the two-stage recruitment process of case/control mothers/relatives in the present study resulted overall in a moderate participation rate of mothers/relatives (50% of the total sample). Very few published studies on TGCT risk factors included case and control mothers. The participation rate of mothers was similar to that of previous studies having included case and control mothers [14, 71–73] and the approach that we used was the one identified as the most promising from the experience of our pilot study [23]. Some differences were observed when comparing participants’ characteristics in groups of interviewed and not interviewed mothers: there were more cases in the interviewed mothers’ group, consequently, the age distribution was different between the two groups and family history of TGCT was reported more frequently in the interviewed mothers’ groups. However, pre- and perinatal factors of participants, and socio-economic statuses of mothers at birth and of participants at interview were similar in the two groups.

Finally, our results may be limited in terms of statistical power, because of the low prevalence for specific exposure variables and small numbers in subgroup analysis, leading to effect estimates with large statistical uncertainty. Despite consistence in main and secondary analyses, we also cannot rule out that our findings were chance due to multiple testing.

## Conclusions

Early life exposures to environmental factors acting as EDC, including pesticides, have been suspected to increase TGCT risk later in life. Our study confirmed that in France parental domestic use of pesticides during early life periods was very common in the general population. Although recall bias is likely, our study suggests that domestic use of fungicides, use of fungicides and/or insecticides for woodwork and use of insecticides on pets during early periods of development may increase the risk of adult TGCT and NS in the offspring. Investigating EDC exposures from multiple sources and during critical periods of development, including preconception, pregnancy and puberty, may be most relevant for future research on TGCT occurrence.

## Supplementary Information

**Additional file 1.** Supplemental Excel File (Tables S1 to S11 and Figure S1).

## Data Availability

The datasets used and/or analysed during the current study are available from the corresponding author on reasonable request.

## Abbreviations

ART: Assisted reproduction treatment
CECOS: *Centres d’étude et de conservation des oeufs et du sperme*
CI: Confidence interval
DDE: p,p’-dichlorodiphenyldichloroethylene
EDC: Endocrine disrupting chemical
GIS: Geographic Information System
IARC: International Agency for Research on Cancer
ICD-O: International Classification of Disease for Oncology
ISCO: International Standard Classification of Occupations
GCNIS: Germ cell neoplasia in situ
HCB: Hexachlorobenzene
NAF: *Nomenclature d’activités française*
NS: Non-seminomas
OR: Odds ratio
SAS: Statistical Analysis System
SE: Seminomas
TGCT: Testicular germ cell tumours
WHO: World Health Organization.
